# Evaluation the Surface Antigen of the *Salmonella typhimurium* ATCC 14028 Ghosts Prepared by “SLRP”

**DOI:** 10.1155/2014/840863

**Published:** 2014-03-19

**Authors:** Amara A. Amro, Ahmed J. Neama, Ahmed Hussein, Emad A. Hashish, Salah A. Sheweita

**Affiliations:** ^1^Department of Protein Research, Genetic Engineering and Biotechnology Research Institute, City for Scientific Research and Technology Applications, New Borg Al-Arab, P.O. Box. 21934, Alexandria, Egypt; ^2^Biotechnology Department, Institute of Graduate Studies & Research, Alexandria University, 163 El Horreya Avenu, P.O. Box 832, Alexandria 21526, Egypt; ^3^Zoonoses Research Unit, Faculty of Veterinary Medicine, Alqadisya University, Alqadisya, Iraq; ^4^Department of Clinical Pathology, Faculty of Veterinary Medicine, Zagazig University, Zagazig, Egypt

## Abstract

Recently, bacterial ghosts (BGs) were prepared using a protocol based on critical chemical concentrations. It has been given the name “sponge like” (SL) protocol and used in its reduced form “sponge like reduced protocol” (SLRP). While specific antibody for * Salmonella* is available on the market under the commercial names (of some kits) such as Febrile Antigen Kit (N.S. BIO-TEC), we used the described Kit to investigate the validity of the SLRP. In this study, using SLRP we succeeded to prepare STGs with correct surface antigens could interact with their specific antibodies. Additionally the study has included oral vaccination with STGs with challenge test. The rats serums have been evaluated against both of the O and H antigens. The antigen-antibody interaction (agglutination) results of both the SLRP and the animal experiments prove that we have correct STGs able to immunize the rats against viable * Salmonella*. STGs could be used as vaccine and as adjuvant and in the antibodies and in the diagnostic kits production. This study is an additional step for the establishment of correct BGs for immunological purposes.

## 1. Introduction

BGs are dead cells without their DNA, protein, and the other cytoplasmic constituents. BGs are a wonderful tool for preparing different kind of bacterial vaccines [1]. That is because they are actually dead empty cells with correct 3D structure. After removing their cytoplasm contents, the cells will not be able to replicate any more. For that, upon entering to our bodies or to the animal's bodies, they will be able to induce the immune system to produce the appropriate antibodies safely. However, the quality of the BGs is a critical issue. Nowadays the common use of the BGs as drug delivery system or in stimulating the immune system [1–6]. The well-established protocol is based on a cloned *E lysis* gene (a bacteriophage ΦX174 gene). The* E lysis *gene is responsible for the phage release from* E. coli* [7]. After lysis, DNA nuclease is used to degrade any existing genetic materials could code for a pathogenic protein for safer BGs preparation (for different applications) [4, 8, 9]. Recently Amara et al. [4–6] have described a new protocol for BGs preparation based on the critical concentration of chemical compounds could effect on the bacterial cell wall and could lead to the preparation of correct BGs [4–6]. The unique criteria of such protocol are that no genetically modified or recombinant elements were used but only the bacterial cells and the described chemical compounds were used. Such protocol is considered safe for the preparation of the BGs. The critical concentration as well as the protocol steps lead to killing the bacterial cells with the release of their cytoplasmic constituents without the deformation of the cell 3D structure [4–6]. For more details, refer to Amara et al. [4–6]. The original protocol has been given the name “Sponge Like” while, after causing minor pores in the bacterial cells, the further steps and particularly the centrifugation steps cause the release of the cytoplasmic constituents by pressing the cells gently like a sponge to release their content to the surrounding environment. In this study, we are adding a further step to the protocol, which is dealing with the cell wall surface antigens, where* Salmonella typhimurium* ATCC 14028 was used for such purposes. The different antibodies for* Salmonella *are available in the market and correct STGs have a positive agglutination result upon their interaction with the antibodies. In addition, the antibodies produced by the STGs should react with the evaluable antigens (O and H) and the immunized animals should be survived after treatment with live cells.

## 2. Material and Methods

### 2.1. Bacterial Strain

*S. typhimurium* ATCC 14028 strain was used in this study and has been kindly provided by the Department of Microbiology, Faculty of Veterinary, Cairo University, Cairo.

### 2.2. Bacterial Cultivation

The* S. typhimurium* ATCC 14028 was cultivated in 500 mL NB (in one-liter flask) under static condition at 37°C for 72 hrs.

### 2.3. Determination of the MIC and MGC for NaOH, SDS, and H_2_O_2_

The MIC and MGC for each of NaOH, SDS, and H_2_O_2_ were determined using standard criteria [4–6, 10]. 10% of each of the NaOH and SDS as well as 30% H_2_O_2_ stock solution was prepared. Standard serial dilution method was used to determine MIC and MGC values, where 0.5 mL of the above solutions were added (each) to 4.5 mL in 10 mL test tube (to gain the final volume 5 mL and the 1 : 10 dilution), and so on. 100 *μ*L of an overnight* S. typhimurium* culture was added to each tube in the serial dilution experiment. In case of CaCO_3_ the used amount of +1 value was 1.05 *μ*g/mL, while −1 value was 0.35 *μ*g/mL. The test tubes were then incubated overnight at 37°C under static cultivation condition.

### 2.4. Sponge Like Reduced Protocol for Preparing the BGs

Two experiments with different parameters (as in Table 1) were conducted. The two experiments were selected from the results obtained by Amara et al. [4] for the best conditions for BGs preparation. They are mainly experiments numbers one and eleven in the original SL protocol [4], and as described in the Sponge Like Reduced Protocol [6] (Table 1).

### 2.5. Determination of the DNA and Protein Concentrations

The concentrations of the DNA and the protein were derived from the spectrophotometer measurement (Thermo Scientific-VISIONpro Software V4.30) and determined according to Amara et al. [4].

### 2.6. STGs Evaluation Using Light Microscope (Optika)

Bacterial smears for both preparations (experiments 1 and 2 as in Table 1) were stained using crystal violet for 30 min. The cells were examined using the light microscope. The cells' quality was determined for each preparation based on the quality of the 3D structure as either being correct or deformed. The overall BGQ for each was given as a %. The images were collected using the camera (MICROS Cam) and the Microsoft image analysis provided (MICROVISIBLE v 1.11.10).

### 2.7. Sample Preparation for Electron Microscope Examination

Electron microscope was used to evaluate the bacterial cells 3D structure. The bacterial smears were prepared using glutaraldehyde standard method for fixation. The dry bacterial smears were coated with approximately 15 nm gold (JEC-1100 E Sputter Coater).

### 2.8. Scanning of the STGs Surface

The golden coated samples then were scanned using scanning electron microscope (JEOL JSM-5300). The secondary element was at 30 kv acceleration voltage (at room temperature). The digital images of the samples were then adjusted and saved for further investigation.

### 2.9. Viability Test

The prepared STGs were evaluated for the existence of any still viable cells, where samples were taken from each preparation and grow in S-S and/or XLD agar plates. The plates then were incubated (in the incubator) at 37°C for two days.

### 2.10. Agglutination Test for STGs Surface Antigen-Antibodies Reaction

Febrile Antigen Kit (N.S. BIO-TEC) was used for Rapid Slide test for the qualitative determination of specific antibodies present in serum against* Salmonella* [11, 12]. The antibodies in the Kit were used to react with the* S. typhimurium* ATCC 14028 wild and STGs. The Positive Control prepared from a stabilized human serum pool with clear agglutination at titer more than 1/80. All components contain 0.l% sodium azide as preservative. Viable* S. typhimurium* ATCC 14028 used in this study was used as a control as well as the antigen (O-antigen of* Salmonella*) provided by the Kit. Each of the wild strain (control 1), the Kit O-antigen of* Salmonella *(control 2), STGs from experiment 1, and STGs from experiment 2 were investigated for their ability to induce agglutination with the provided antibodies. The reactions were made on the surface of single slight where five *μ*L of each sample was tested. Then 3 *μ*L of the positive* Salmonella* antisera was added to the previous samples. The slight was gently vibrated to insure mixing each of the four above mixtures. Then the slight was left for four min to ensure complete reactions. The results of the agglutination reaction of the sample were observed with the naked eye. Then the agglutinations of the four samples were investigated under the light microscope. An image from each of the four reactions was take nusing a camera (MICROS Com) and the Microsoft image analysis provided MICROVISIBLE v 1.11.10. The images were saved as JPG files.

**Figure 1:**
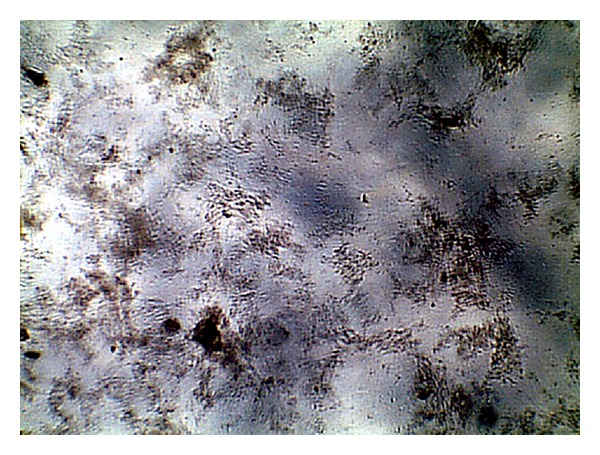
Agglutination reaction of* S. typhimurium* ATCC 14028.

**Figure 2:**
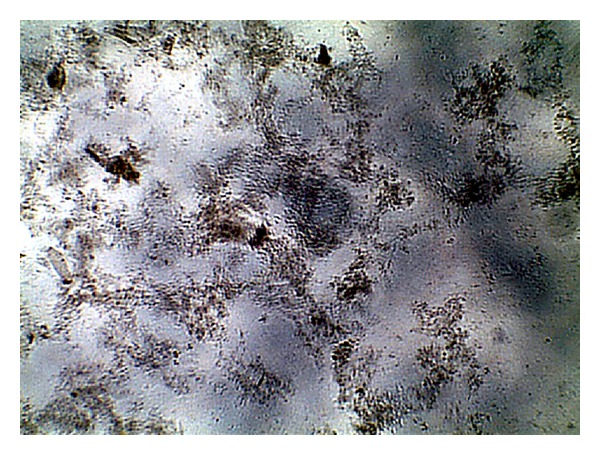
Agglutination reaction of BGs prepared from* S. typhimurium* ATCC 14028 using experiment number 1 as in Table 1.

**Figure 3:**
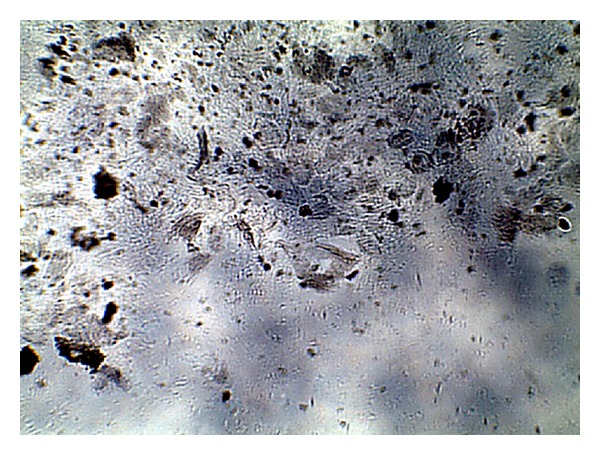
Agglutination reaction of BGs prepared from* S. typhimurium* ATCC 14028 using experiment number 2 as in Table 1.

**Figure 4:**
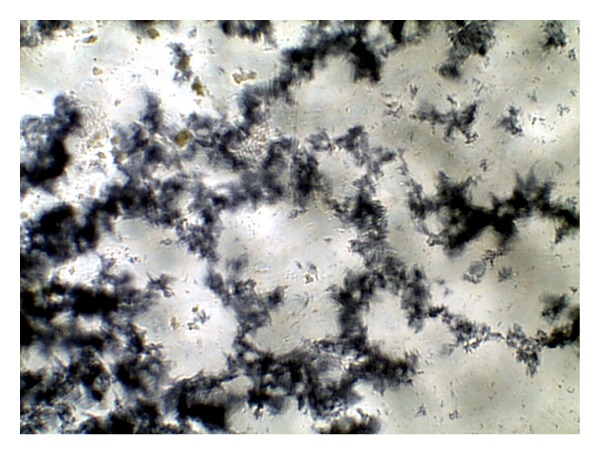
Agglutination reaction of standard O-antigen.

The images were collected as JPG image as shown in Figures 1, 2, 3, and 4. For more details about the Kit constituents and procedure refer to the Kit manual.

### 2.11. Rats

Ten male Sprague-Dawley albino rats (200 gm ± 5 gm) were housed in animal house of the Institute of Graduated Studies and Research (Alexandria, Egypt) and used in this study for oral vaccination. They were divided into two groups with each containing five animals. The first group was used as control while the second group has received oral dosage of STGs and Freund's adjuvant. The rats were housed in a cage, according to NIH guidelines with free access to distilled water and food.

### 2.12. Oral Vaccination and Challenge Test

The rats (6-week old) in group two received each a mixture of 70 *μ*g of STGs/100 *μ*L saline mixed with 100 *μ*L of complete Freund's adjuvant. After two weeks, the animals received 70 *μ*g of STGs/100 *μ*L saline mixed with 100 *μ*L of incomplete Freund's adjuvant. After further two weeks, the animals received only 70 *μ*g of STGs/100 *μ*L saline without adjuvant. Serum from each animal was collected from the vain in the tail using standard criteria before each treatment. After one week, each animal has received 10^8^ CFU of live* Salmonella typhimurium* ATCC 14028 once. Samples from the treated animal from the faces were examined by SS-agar medium through one week following the first day of challenge experiment. The antibodies of the serum were tested using the previous described agglutination test using both of the O and the H antigens. The data were summarized in Table 1. Animals were slighted after 7 days, and their livers were collected, and homogenized under aseptic conditions and sample from each was cultivated on the SS-agar medium.

## 3. Results and Discussions

BGs new protocol was recently published [4–6]. The protocol describes a simple method for BGs preparation using critical chemical concentration enable the remove of the bacterial cytoplasmic constituents. Particularly, the MIC and the MGC of each of the used compounds were determined. MIC and MGC determination is critical step in the BGs preparation. Because they are the points, where the bacterial dead or survive. Minimum concentration (inducing cells killing) might not degrade the bacterial cell wall as described by Amara et al. [4], and MGC in the existence of such chemical compounds did not guarantee that the cells are not affected. Life cells could be injured by the concentrations of the used compounds (in case of the MGC). For that and by using several steps in the protocol the bacterial cell loss their content of DNA and Protein. The first point is to expose the bacterial cells to the chemical compounds either alone or in correct combination (did not enable chemical interactions), and the seconded point is to enable several centrifugation and washing steps to remove the DNA and the protein content completely. The gentle centrifugation enables fine pressing to the cells to enable them to get rid of their cytoplasm constituents. The original protocol [4] has used Plackett and Burman [13] for randomizing the variables based on using MIC as the +1 value and used MGC as the −1 value. However, Plackett-Burman experiments lead to a long protocol [13]. For that, Amara et al. [6] reduced the original protocol. The concept has become clear that MIC and MGC are the most crucial factors in the BGs preparation and the protocol was improved significantly. However, it was still an open scientific question: did the BGs preparation using such protocol enable good surface antigen could activate the immune system? For that, in this study, we get the benefit from the existence of commercial kits that enable the detection of the presence of the* Salmonella* antigens to evaluate the BGs prepared from* S. typhimurium*. The MIC and the MGC for each of NaOH, SDS, and H_2_O_2_ were determined, respectively. The tubes, which were incubated (for overnight), were observed and both of the MGC and the MIC for each treatment were determined. In case of the NaOH and the SDS, the MIC and the MGC were 0.1 mg/mL and 0.01 mg/mL, respectively. In case of H_2_O_2_ the MIC and the MGC were 0.03% and 0.003%. The release of the DNA and the protein were determined spectrophotometrically using quartz curvet at 260  and 280 nm, respectively. The results show correct release of both of the DNA and the protein during the preparation steps. The amount of the protein is greater than that of the DNA as shown in Table 1. That is logic, particularly at the H_2_O_2_ step. None of the cells in each of the preparation upon cultivation on NA medium for two days at 37°C has shown any growth.

**Figure 5:**
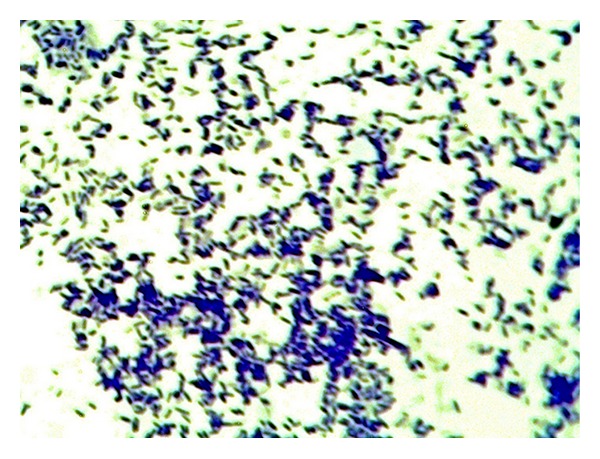
Light microscope for the STGs. The cells show correct 3D structure.

**Figure 6:**
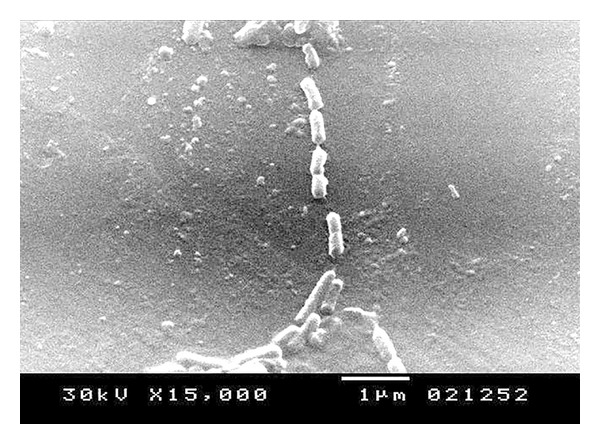
Electron microscope for the STGs at 15000x.

**Figure 7:**
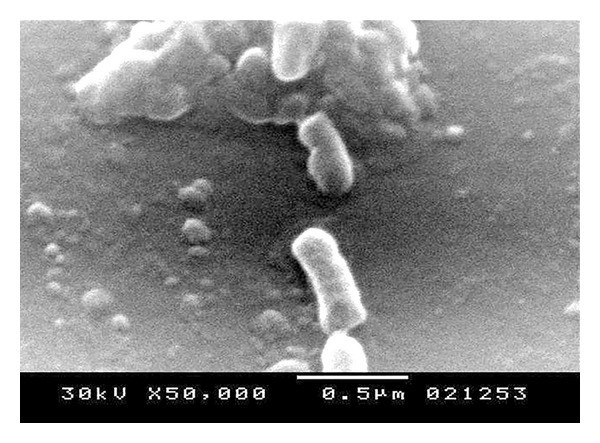
Electron microscope for the STGs at 50000x.

**Figure 8:**
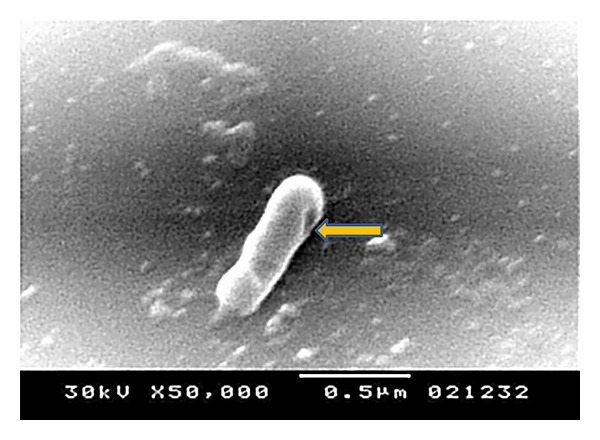
Electron microscope for the STGs at 50000x. The arrow shows a single pore in the cell.

**Figure 9:**
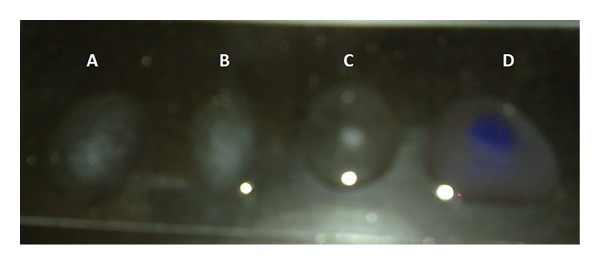
Agglutination experiment all samples contain antibodies plus (A)* S. typhimurium* ATCC 14028, (B) STGs from experiment 1, (C) STGs from experiment 2, and (D) the kit control (O antigen).

The quality of the prepared BGs (QBGs) were monitored using both of the light and the electron microscope. Both show the correct 3D structure of the cells as in Figures 5, 6, 7, and 8. After being sure from the preparation quality (BGQ as in Table 1), the agglutination test was conducted using Febrile Antigen Kit (N.S. BIO-TEC). The Kit contains the antibodies and controls to validate the antigens and the antibodies. The agglutination test was used following the standard criteria and the steps described in the Kit user manual. The result shows clear agglutination in the* S. typhimurium* wild type and the BGs prepared from experiments one and two as well as the kit control as in Figure 9. Additionally, the various agglutinations were tested using the 10x lens of the light microscope as shown in Figures 1–4. That proves the existence of correct antigens on the surface of the BGs prepared using this protocol.

The oral vaccination test shows positive interaction as in Table 2. In the first two weeks the H antigen shows weak agglutination with the serum. The O antigen shows positive result (agglutination within one minute). The other reactions in Table 2 concerning group 2 shows all positive antigen-antibody reaction after treatment with the STGs. Group 1 shows negative results in all cases. The data obtained from the challenge test, evaluated by the cultivation of samples from the animal feces as well as from the liver homogenate after the challenging test, show no growth for the* Salmonella typhimurium* ATCC 14028. None of the STGs treated animals were dead after the challenge test. Only one rat from the control (receive viable* Salmonella*) has been dead after the challenge test. Apparently, from the qualitative data of the antigen-antibody interaction, the rats in group 2 were well immunized against each of the O and H antigens of the* Salmonella typhimurium* ATCC 14028. The challenge test shows the ability of the animal to survive after using live bacterial dosage and show ability to cure the* Salmonella* cells from both of the feces which represent correct anti-Salmonella activity, as well as, there were no cells growth from liver homogenate. Samples STGs prove to be efficient for immunizing the rats, production of antibodies, correct surface antigen, and correct 3D structure as proved by the results obtained from the electron microscope Figures 6–8. The tactic of using Freund's adjuvant in the oral treatment, even not new, proves to be efficient. For similar oral use for Freund's adjuvant in oral treatment, refer to [14–22]. We did not decrease the amount of the antigens (STGs) after the first treatment while STGs are not living cells, and we use oral treatment where the antigens are in minor contact with the blood. However, oral vaccination is an additional proof for the efficacy of STGs surface antigens validity to induce correct immunization.

This study has taken an advantage from the evaluable* Salmonella* diagnostic kits evaluable in the market to shorten the experiment times. However, it rehighlights the importance of Widal test. STGs prepared using the new protocol might be a product in the coming future for vaccination, in diagnosis, and antibodies production, or even new.

In this study, we prove for the first time the validity of the BGs prepared using our new protocol to show correct antigen-antibodies interaction and its suitability for any of the immunological processes and their related applications such as preparation of vaccines, adjuvant, and bacterial surface antigens.

## 4. Conclusion

This study, for the first time, describes a method for preparation of BGs from* Salmonella typhimurium* ATCC 14028 using the MIC and the MGC of SDS, NaOH, and H_2_O_2_ and a selected concentration for CaCO_3_ as described in the SLRP. The study describes for the first time the validity of the surface antigen of the BGs after their preparation and their ability to be used as vaccine. The oral vaccination for rats using the STGs and Freund's adjuvant proves protection for the animals against the viable* Salmonella* during the challenge test. The serum (after vaccination) is able to agglutinate with both of O and H antigens of the* Salmonella*. This study is an additional step toward improving the quality and the validity of the BGs prepared using such protocol and a step toward immunological products from BGs prepared by either SL or SLRP. STGs prepared in this study can be used as vaccine, adjuvant and in* Salmonella* diagnostic kits.

## Figures and Tables

**Table 1:** Experiments one and two for BGs preparation.

Experiment no.	Experiment variables					Basic Experiment		H_2_O_2_ Step		Ethanol step		BGs quality as % (BGQ)
	NaOH	CaCO_3_	H_2_O_2_	SDS	Shaking rate-Temperature	Protein µg mL^−1^	DNA µg mL^−1^	Protein µg mL^−1^	DNA µg mL^−1^	Protein µg mL^−1^	DNA µg mL^−1^	
1	−1	1	1	1	1	3520.8	109.5	3535.47	150	3286	139	100
2	−1	−1	−1	1	1	6425.46	265	4063.59	339	4122	203	90

**Table 2:** Different agglutination results of the collected serums.

Agglutination test time	Antigen type	Group 1	Group 2
Before first treatment	O	−	−
	H	−	−

Before second treatment	O	−	+
	H	−	+*

Before third treatment	O	−	+
	H	−	+

Before Challenge	O	−	+
	H	−	+

*Weak agglutination.
